# 2073. Impact of in-house *Candida auris* polymerase chain reaction (PCR) screening on admission on the incidence rates of surveillance and clinical cultures with *C. auris* and associated cost-savings

**DOI:** 10.1093/ofid/ofad500.143

**Published:** 2023-11-27

**Authors:** Rossana M Rosa, Adriana Jimenez, David Andrews, Huy Dinh, Katiuska Parra, Octavio Martinez, Lilian M Abbo

**Affiliations:** UnityPoint Health, Urbandale, IA; Jackson Health System, Infection Prevention and Control Department, Miami, FL; University of Miami, Miami, Florida; Jackson Health System, Miami, Florida; Jackson Health System, Miami, Florida; Jackson Health System/University of Miami, Miami, FL; University of Miami Miller School of Medicine, Miami Transplant Institute and Jackson Health System, Miami, FL

## Abstract

**Background:**

Candida auris (*CA*) transmission has significantly increased in the United States. Routine screening by PCR methods and economic impact have not been previously defined. We present our experience implementing in-house PCR testing on admission for screening of colonization with *C*A in an area of high prevalence.

**Methods:**

Study conducted across the acute care and inpatient rehabilitation hospitals at a large integrated, university affiliated, public health system in Miami, Florida. The pre-intervention (August 1, 2019 -July 31, 2021) testing was sent out to a refence laboratory, and post-intervention (August 1, 2021 - January 31, 2023) testing was performed in-house. Our screening, testing and isolation protocols are presented in Figure 1A and 1B. Interrupted time series was done to analyze changes in the incidence rates of CA present on admission (CA-POA) and hospital-onset fungemia (CA-HOF), and the estimated economic impact on isolation costs and testing were calculated.

Candida auris screening and isolation process

**Figure:**
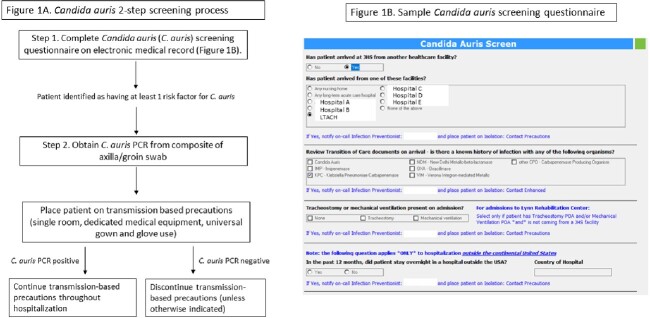

Figures describing the 2-step screening process for Candida auris, including the questionnaire on the electronic medical record, and the transmission-based precautions followed

**Results:**

A total of 4,478 PCRs were done on 4,270 patients. We identified 159 unique CA-POA. During the pre-intervention period there was a baseline trend towards a monthly increase in the incidence rate of CA-POA of 3% (95% CI 1.00-1.06; p=0.11). Following the intervention there was an immediate change with an incidence rate ratio of 2.57 (95% CI 1.16-5.69, p=0.02) compared to the pre-intervention period (Figure 2A). Seventy-five unique cases of CA-HOF were identified. In the pre-intervention period, the baseline rate of CA-HOF was increasing monthly by 14% (95% CI 1.05-1.24; p=0.002). No immediate change in rates was seen after introducing in-house testing; throughout the post-intervention period there was a change in slope showing a monthly decrease in rates of 13% (95% CI 0.80-0.99; p=0.02) (Figure 2B). A preliminary economic impact analysis is presented in Table 1.

Interrupted Time Series Analysis of rates of Candida auris present on admission and Candida auris hospital-onset fungemia

**Figure:**
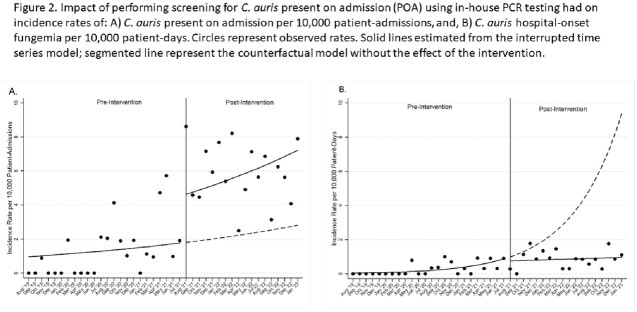

Impact of performing screening for C. auris present on admission (POA) using in-house PCR testing had on incidence rates of: A) C. auris present on admission per 10,000 patient-admissions, and, B) C. auris hospital-onset fungemia per 10,000 patient-days. Circles represent observed rates. Solid lines estimated from the interrupted time series model; segmented line represent the counterfactual model without the effect of the intervention.

**Figure:**
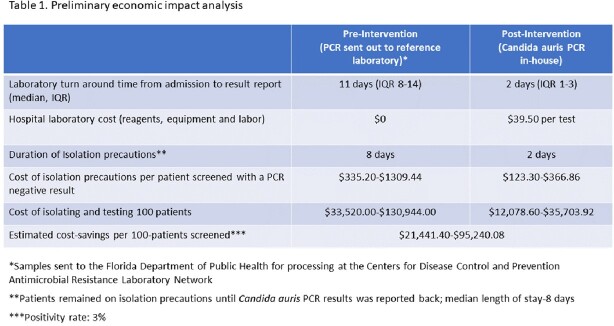

Table summarizing the costs and savings associated with the intervention

**Conclusion:**

By performing in-house PCR colonization screening on admission for the detection of CA, we found a doubling of CA-POA rates, and a subsequent decrease in the incidence rates of CA-HOF. In-house testing was cost effective in our setting, with savings estimates ranging from $21,441.40 to $95,240.08, and should be considered in areas of high prevalence to decrease invasive infections and prevent horizontal transmission.

**Disclosures:**

**Lilian M. Abbo, MD, MBA**, Ferring: Advisor/Consultant|Pfizer: Advisor/Consultant|Regeneron: Grant/Research Support|Shionogi: Advisor/Consultant

